# Association between death literacy and life-sustaining treatment preferences in patients with advanced non-small cell lung cancer

**DOI:** 10.1016/j.apjon.2026.100987

**Published:** 2026-06-05

**Authors:** Gong-Fu Lin, Ching-Yao Yang, Yi-Chun Chen, Yi-Ling Yeh, Shiow-Ching Shun, Cheng-Pei Lin

**Affiliations:** a Department of Nursing, National Taiwan University Hospital, Taipei, Taiwan; b Department of Internal Medicine, National Taiwan University Hospital, Taipei, Taiwan; c Department of Social Work, Tunghai University, Taichung, Taiwan; d Institute of Clinical Nursing, College of Nursing, National Yang Ming Chiao Tung University, Taipei, Taiwan; e Institute of Community Health Care, College of Nursing, National Yang Ming Chiao Tung University, Taipei, Taiwan; f Cicely Saunders Institute, Florence Nightingale Faculty of Nursing, Midwifery and Palliative Care, King's College London, London, United Kingdom

**Keywords:** Lung cancer, Death literacy, Life-sustaining treatment, Preference

## Abstract

**Objective:**

Evidence suggests that individuals with higher death literacy levels are more capable of planning their own end-of-life care. Lung cancer has the highest mortality among all cancers; therefore, early clarification of preferences for life-sustaining treatments (LSTs) represents an important and urgent clinical concern. This study aimed to examine the association between death literacy and preferences for LSTs among patients with advanced non–small cell lung cancer (NSCLC).

**Methods:**

This cross-sectional study included 103 patients with stage IIIB or higher NSCLC at a medical center in northern Taiwan. Data were collected using structured questionnaires that assessed patients’ sociodemographic characteristics, death literacy, and life-sustaining treatment preferences. Descriptive statistics were used to summarize participant characteristics. Pearson’s correlation was used to analyze associations between LST preferences and death literacy. One-way ANOVA was used to analyze differences in preferences for LSTs by tertiles of total death literacy scaled mean scores: low (≤ 5.21), moderate (> 5.21 to ≤ 6.59), and high (> 6.59).

**Results:**

Among the LST options, cardiopulmonary resuscitation (CPR) was rated as the least desirable, whereas antibiotic therapy was the most favored. Higher overall death literacy scores, as well as the subscales of practical, experiential, and factual knowledge in death literacy, were significantly and inversely associated with preferences for LSTs, such as antibiotics, blood transfusion, hemodialysis, nasogastric tube insertion, intubation, and CPR; this indicated a modest correlation with lower willingness to receive these treatments. In addition, compared with the low death literacy group, patients in the high death literacy group were less likely to prefer aggressive LSTs.

**Conclusions:**

Patients with higher death literacy levels were less likely to prefer aggressive LSTs. This study provides an initial empirical foundation for clarifying the nature and direction of these associations. Future research with larger and more diverse samples, as well as with longitudinal designs, is warranted to examine how death literacy and treatment preferences evolve over time across different disease populations.

## Introduction

Lung cancer is the leading cause of cancer deaths worldwide,^1^ with non–small cell lung cancer (NSCLC) accounting for approximately 85% of all diagnoses.^2^ Despite advances in targeted therapies in recent decades, patients with advanced NSCLC continue to have poor prognoses,^3^ with overall 5-year survival rates ranging from 0% to 10% among those with stage IVA–IVB disease.^4^ According to the 2024 Taiwan Cancer Registry, lung cancer accounted for 10,495 cancer-related deaths, making it the leading cause of cancer mortality in Taiwan.^5^ Mortality in patients with lung cancer is commonly attributable to tumor burden, infections, metastatic complications, pulmonary parenchymal hemorrhage, pulmonary embolism, and diffuse alveolar damage.^6^ Compared with patients with other malignancies, patients with NSCLC tend to experience more severe symptom burden and greater functional impairment.^7^ Accordingly, early identification of end-of-life (EOL) care preferences in this population is both important and urgent.^8^

With growing recognition of the value of EOL care, attention has increasingly shifted toward aligning care with individual preferences. This is regarded as a key indicator of high-quality EOL care and as essential for delivering value-concordant care.^9^^,^^10^ However, high-quality advanced lung cancer care involves the early integration of palliative care services and enhancement of death literacy.^11^ Evidence suggests that individuals with higher levels of death literacy are more capable of planning their own EOL care.^12^ Patients with advanced cancer frequently encounter complex decisions regarding life-sustaining treatments (LSTs).^13^ Clarifying preferences for LSTs constitutes a fundamental component of discussions surrounding EOL care goals.^14^ For patients with terminal illness, for whom the disease is incurable and death is inevitable, aggressive LSTs often serve primarily to prolong the dying process, potentially increasing suffering for both patients and their families.^15^ Decision-making related to LSTs is particularly complex in East Asian contexts, where family involvement and cultural norms play a pivotal role.^10^ Taiwanese society is generally characterized as a high-context culture in which discussions about death are often avoided, and decision-making tends to be family- rather than individual-centered. Such cultural dynamics may hinder autonomous decision-making regarding preferred EOL care.^10^

Death literacy is an emerging concept introduced by Noonan and colleagues in 2016, and it is defined as a set of knowledge and skills that enables individuals to access, understand, and act upon EOL and death-related information.^16^ As a practical framework, death literacy provides individuals with resources to support their decision-making and strengthen their capacity for future caregiving.^12^ Death literacy mirrors the concept of health literacy.^17^ Previous research has primarily focused on how health literacy is associated with EOL care preferences and decision-making.^18^^,^^19^ Health literacy refers to the cognitive and social skills required to obtain, understand, and use health-related information to manage one’s health condition.^20^ Lower health literacy levels have been shown to be associated with a greater likelihood of choosing aggressive LSTs.^18^ The role of death literacy, an extension of health literacy that specifically encompasses knowledge and competencies related to death, dying, and EOL care,^16^ remains insufficiently explored. Therefore, this study aimed to examine the association between death literacy and preferences for LSTs among patients with advanced NSCLC.

## Methods

### Study design

This study was part of a larger research project and utilized the same dataset as our previously published article.^21^ However, the present analysis addresses different research aims and hypotheses and examines distinct analytical outcomes. Specifically, this study focuses on the association between death literacy and preferences for LSTs, using a cross-sectional descriptive correlational design. The Strengthening the Reporting of Observational Studies in Epidemiology (STROBE) checklist was used for reporting.^22^

### Study participants

We recruited patients aged ≥ 18 years who had a histologically confirmed diagnosis of NSCLC, were at stage IIIB or higher, and had completed at least one line of cancer treatment. Participants were excluded if they: (1) had primary lung cancer with brain metastases that impaired consciousness, comprehension, or expression; (2) could not communicate or read fluently in Mandarin or Taiwanese; or (3) had been diagnosed with multiple types of cancer, to reduce potential heterogeneity and confounding effects. The required sample size was estimated using G∗Power version 3.1.9.2 for correlational analysis with *α* = 0.05, power = 0.80, and a medium effect size (*r* = 0.30), yielding a minimum sample size of 84 participants.^23^ After accounting for a potential incomplete response rate of 20%, the minimum required sample size was 102 participants.

### Study setting

The study was conducted in a 37-bed chest medicine ward of a national medical center in northern Taiwan. The medical center has more than 2300 beds and over 7500 staff members, provides a comprehensive range of specialty and subspecialty services, and is one of the largest hospitals in northern Taiwan.

### Data-collection process

Data were collected between June 2024 and June 2025. Eligible patients in the Chest Medicine ward were screened by reviewing their electronic medical records, which informed the treating attending physician of potential candidates. The treating attending physician then verbally invited eligible patients to participate in the study. Written informed consent was obtained from all participants prior to scheduling the interviews. We conducted one-to-one, face-to-face interviews, which allowed us to reduce missing data and mitigate incomplete questionnaire content.^24^ Data collection was conducted using a structured questionnaire, which participants typically completed independently. In cases where participants were unable to read the questionnaire, items were read aloud, and the participants’ responses were documented by the researcher. A voucher equivalent to NTD 200 (approximately USD 7) was provided to the participants as a token of appreciation for their time.

### Measurements

#### Demographic characteristics

The demographic characteristics consisted of three main sections: (1) demographic characteristics, (2) disease-related characteristics, and (3) prior firsthand experiences related to EOL care. Demographic variables included age, sex, educational attainment, financial status, and religious affiliation. Disease-related variables encompassed the time since diagnosis, Charlson Comorbidity Index (CCI), and Eastern Cooperative Oncology Group (ECOG) performance status. Prior firsthand EOL care experiences captured whether participants had previously engaged in EOL care discussions with a physician, provided EOL care to others, or experienced the death of a relative. A total of 12 independent variables were assessed using this form.

#### Death literacy index (DLI)

Death literacy was measured using a traditional Chinese version translated by Yeh in 2022,^25^ who had previously recruited 708 community participants for the validation of the DLI. Confirmatory factor analysis (CFA) demonstrated an acceptable model fit: χ^2^ (369) = 1644.314, *P <* 0.001, Comparative Fit Index (CFI) = 0.913, Non-Normed Fit Index (NNFI) = 0.891, Root Mean Square Error of Approximation (RMSEA) = 0.070, indicating that the revised model fit the observed data well. In addition, the overall Cronbach’s alpha coefficient of the translated DLI was 0.938, while those of the subscales were in the range 0.779–0.939. The traditional Chinese version consists of 29 items and four subscales: practical knowledge (eight items), experiential knowledge (five items), factual knowledge (seven items), and community knowledge (nine items). All items were rated on a five-point Likert scale ranging from 1 (strongly disagree) to 5 (strongly agree). The use of scaled mean scores was adopted to prevent unequal weighting due to variations in the number of items across subscales. Scores are calculated by summing items and scaling the number of items in a subscale (with a range of scores between 0 and 10, with higher total scores indicating greater death literacy). Cronbach’s alpha for the traditional Chinese version of the DLI in this study was 0.920.^21^

#### Life-sustaining treatments preference interview questionnaire

The questionnaire was developed in collaboration with a multidisciplinary expert group that included palliative care physicians, hospice clinical nurse specialists, social workers, nursing scholars, and psychologists. In this study, LSTs encompassed antibiotic therapy, blood transfusion, hemodialysis, nasogastric (NG) tube insertion, endotracheal intubation with mechanical ventilation, and cardiopulmonary resuscitation (CPR). Participants’ preferences for each LST were evaluated independently using a seven-point Likert scale ranging from 1 (strongly disagree) to 7 (strongly agree). Preferences for LSTs and goals for EOL care were elicited through situational questions accompanied by descriptive vignettes. Each vignette provided information on the potential benefits and drawbacks of accepting or refusing a particular treatment. For example, CPR was described as follows: “For individuals with advanced cancer, CPR may not successfully restore the heartbeat and may cause adverse effects such as soreness, rib fractures, or lung collapse.” After the item descriptions and explanatory statements were finalized, five palliative care experts, including physicians, clinical nurse specialists, social workers, nursing scholars, and clinical psychologists, were invited to evaluate the questionnaire for applicability, clarity, and relevance. Content validity was assessed using the content validity index (CVI). The scale-level CVI (S-CVI) was 0.98, indicating excellent content validity. A minimum S-CVI of 0.80 is generally recommended as the acceptable threshold for content validity.^26^

### Statistical analysis

Data were analyzed using IBM SPSS Statistics 30. Descriptive analyses, including means, standard deviations, frequencies, and percentages, were used to summarize participants’ demographic characteristics. The distribution of LST preference scores was considered approximately normal because the skewness and kurtosis values fell within the acceptable range. Therefore, parametric analyses were used.^27^ Pearson’s correlation analysis was used for bivariate analyses of continuous variables. Death literacy was categorized into three groups based on tertiles of the total death literacy scaled mean score (scaled mean score = 5.79 out of 10): low (≤ 5.21), moderate (> 5.21 to ≤ 6.59), and high (> 6.59). One-way ANOVA was performed to examine differences in LST preferences across these three groups. Statistical significance was set at 0.05.

## Results

### Demographics and baseline of LST preference

We approached 110 eligible patients, 103 of whom agreed to participate in the study, with a response rate of 93.6% (four were not interested, and three were too weak to participate). Participants had a mean age of 61.62 ± 10.86 years (range: 34–83 years), and the mean CCI was 7.50 ± 1.95. Among these, 52.4% were men, 83.5% had an ECOG performance status of 0–2, 55.3% had survived for more than 12 months after diagnosis, 59.2% had an education level below senior high school, 80.6% had a financial status ≥ NTD 20,000, 78.6% were in a partnership, and 73% had religious beliefs. Additionally, 58.3% had experience of EOL care provision, and 90.3% had experience of losing relatives; however, only 29.1% had experienced EOL care discussions with a physician. The detailed results are presented in Table 1.

**Table 1:** Demographic characteristics (*N* = 103).

Variable	*n* (%)	Mean (SD)
**Sex**		
Female	49 (47.6)	
Male	54 (52.4)	
**Age, years**		61.62 (10.86)
**Charlson Comorbidity index**		7.50 (1.95)
**Marital status**		
None	22 (21.4)	
With partner	81 (78.6)	
**Educational status**		
Senior high school or below	61 (59.2)	
College or higher	42 (40.8)	
**Financial status**		
< NTD 20,000	20 (19.4)	
≥ NTD 20,000	83 (80.6)	
**Religious belief**		
None	27 (26.2)	
Yes	76 (73.8)	
**Survival after diagnosis**		
> 12 months	57 (55.3)	
≤ 12 months	46 (44.7)	
**ECOG performance status**		
Grade 0-2	86 (83.5)	
Grade 3-4	17 (16.5)	
**Experience of EOL care discussion with a physician**		
None	73 (70.9)	
Has experience	30 (29.1)	
**Experience of EOL care provision**		
None	43 (41.7)	
Has experience	60 (58.3)	
**Experience of bereavement**		
None	10 (9.7)	
Has experience	93 (90.3)	

SD, standard deviation; CCI, Charlson Comorbidity Index; ECOG, Eastern Cooperative Oncology Group; EOL, end-of-life; NTD, New Taiwan dollar.

With respect to LST preferences, participants reported a higher willingness to receive antibiotics (mean = 4.55) and blood transfusion (mean = 4.34), whereas they expressed lower willingness to receive NG tube insertion (mean = 3.05), hemodialysis (mean = 2.88), intubation (mean = 2.26), and CPR (mean = 2.20). The skewness and kurtosis values are presented (Table 2).

**Table 2:** Preferences for LSTs among patients with advanced NSCLC (*N* = 103).

Variable	Mean (SD)	Skewness	Kurtosis
Antibiotic therapy	4.55 (1.85)	−0.27 (0.23)	−1.12 (0.47)
Blood transfusion	4.34 (1.87)	−0.16 (0.23)	−1.01 (0.47)
Hemodialysis	2.88 (2.03)	0.66 (0.23)	−0.87 (0.47)
Nasogastric tube insertion	3.05 (1.95)	0.51 (0.23)	−0.88 (0.47)
Intubation	2.26 (1.79)	1.23 (0.23)	0.38 (0.47)
Cardiopulmonary resuscitation	2.20 (1.74)	1.28 (0.23)	0.62 (0.47)

SD, standard deviation; LSTs, life-sustaining treatments; NSCLC, non–small cell lung cancer.

### Association between death literacy and LST preference

In Table 3, the results display the correlation between death literacy and LST preference among the participants. Higher death literacy was significantly associated with lower willingness to receive all six types of LSTs, including antibiotics (*r* = – 0.402, *P <* 0.001), blood transfusion (*r* = – 0.417, *P <* 0.001), hemodialysis (*r* = – 0.408, *P <* 0.001), NG tube insertion (*r* = – 0.422, *P <* 0.001), intubation (*r* = – 0.529, *P <* 0.001), and CPR (*r* = – 0.492, *P <* 0.001). Higher scores on the practical, experiential, and factual knowledge subscales were significantly associated with lower willingness to receive all six types of LSTs. Practical knowledge showed negative correlations with preferences for antibiotics (*r* = – 0.397, *P* < 0.001), blood transfusion (*r* = – 0.393, *P* < 0.001), hemodialysis (*r* = – 0.312, *P* < 0.001), NG tube insertion (*r* = – 0.377, *P* < 0.001), intubation (*r* = −0.455, *P* < 0.01), and CPR (*r* = −0.426, *P* < 0.001). Experiential knowledge was similarly associated with lower preferences for antibiotics (*r* = – 0.470, *P* < 0.001), blood transfusion (*r* = – 0.462, *P* < 0.001), hemodialysis (*r* = – 0.424, *P* < 0.001), NG tube insertion (*r* = – 0.481, *P* < 0.001), intubation (*r* = – 0.578, *P* < 0.001), and CPR (*r* = – 0.549, *P* < 0.001). Factual knowledge was negatively correlated with preferences for all six LSTs, with relatively weaker associations observed for antibiotics (*r* = – 0.269, *P* < 0.01) and blood transfusion (*r* = – 0.305, *P* < 0.01), compared with those for hemodialysis (*r* = – 0.368, *P* < 0.01), NG tube insertion (*r* = – 0.328, *P* < 0.001), intubation (*r* = – 0.410, *P* < 0.001), and CPR (*r* = – 0.392, *P* < 0.001). In contrast, no statistically significant correlations were observed between community knowledge and LST preferences.

**Table 3:** Association between death literacy and LSTs preferences (*N* = 103).

Variable	M (SD)	A	B	C	D	E	F
DL	5.79 (1.42)	−0.402^b^	−0.417^b^	−0.408^b^	−0.422^b^	−0.529^b^	−0.492^b^
Practical	6.09 (2.01)	−0.397^b^	−0.393^b^	−0.312^a^	−0.377^b^	−0.455^b^	−0.426^b^
Experiential	7.54 (2.00)	−0.470^b^	−0.462^b^	−0.424^b^	−0.481^b^	−0.578^b^	−0.549^b^
Factual	5.80 (1.88)	−0.269^a^	−0.305^a^	−0.368^b^	−0.328^b^	−0.410^b^	−0.392^b^
Community	3.74 (1.27)	−0.027	−0.065	−0.120	−0.047	−0.131	−0.085

LSTs, life-sustaining treatments; DL, death literacy; M, scaled mean scores; SD, standard deviation; A, Antibiotic therapy; B, blood transfusion; C, hemodialysis; D, nasogastric tube insertion; E, intubation; F, cardiopulmonary resuscitation.

a *P* < 0.01.

b *P* < 0.001.

### Differences in LST preferences by death literacy level

In Table 4, significant group differences in preferences for LSTs were observed across death-literacy levels, including antibiotics (*P* < 0.001), blood transfusion (*P* < 0.001), hemodialysis (*P* < 0.001), NG tube insertion (*P* = 0.002), intubation (*P* < 0.001), and CPR (*P* < 0.001). Scheffé post hoc tests further indicated that participants with low death literacy reported a significantly greater willingness to receive these treatments compared with those with high death literacy across all LST items.

**Table 4:** Differences in LSTs preferences by death literacy level (*N* = 103).

Variable	ⓐLow DL (M ± SD)	ⓑModerate DL (M ± SD)	ⓒHigh DL (M ± SD)	*P* value	Scheffé post hoc
A	5.43 (1.44)	4.50 (1.89)	3.71 (1.81)	< 0.001	ⓐ > ⓒ
B	5.20 (1.41)	4.32 (1.91)	3.47 (1.89)	< 0.001	ⓐ > ⓒ
C	3.97 (1.93)	2.62 (2.03)	2.03 (1.64)	< 0.001	ⓐ > ⓑ, ⓐ > ⓒ
D	3.91 (1.82)	2.88 (1.93)	2.32 (1.80)	0.002	ⓐ > ⓒ
E	3.31 (1.90)	2.03 (1.67)	1.41 (1.18)	< 0.001	ⓐ > ⓑ, ⓐ > ⓒ
F	3.11 (1.82)	2.03 (1.69)	1.44 (1.23)	< 0.001	ⓐ > ⓑ, ⓐ > ⓒ

cutoffs: Low DL (≤ 5.21; *n* = 35), Moderate DL (> 5.21 to ≤ 6.59; *n* = 34), and High DL (> 6.59; *n* = 34).

A, Antibiotic therapy; B, blood transfusion; C, hemodialysis; D, nasogastric tube insertion; E, intubation; F, cardiopulmonary resuscitation; M, mean preference score; DL, death literacy; SD, standard deviation; LSTs, life-sustaining treatments.

## Discussion

To the best of our knowledge, this study is the first to specifically examine the association between death literacy and preferences for LSTs. In this study, the distribution of mean scores across the six LSTs among patients with NSCLC revealed a distinct trend, allowing these preferences to be categorized into three value-based groups: high-preference treatments (antibiotics and blood transfusion); moderate-preference treatments (NG tube insertion); and low-preference treatments (hemodialysis, intubation, and CPR). Among the LST options in this study, CPR was rated as the least desirable, whereas antibiotic therapy was rated as the most acceptable.

This preference pattern is consistent with previous findings in Taiwanese and other East Asian contexts. For example, Lin and colleagues^10^ reported that patients with advanced cancer in Taiwan considered a frail disease stage as an indicator for withdrawing invasive LSTs, such as CPR and intubation, except for nutrition and fluid supplementation, antibiotics, and blood transfusions, which tended to be continued. Similarly, Ke and colleagues^28^ indicated that requests for intravenous fluid administration were common among hospitalized patients in Chinese societies, and that older adults in Taiwan tended to favor antibiotic use at EOL, often perceiving these treatments as anti-inflammatory or protective. In addition, from a treatment-modality perspective, both antibiotics and blood transfusions are administered through intravenous access. Compared with aggressive interventions such as CPR, intubation, or hemodialysis, these treatments are generally perceived as less invasive and are associated with lower procedural risks and harm, which may partly explain their higher acceptability.^10^^,^^28^^,^^29^

A notable observation in this study was that overall death literacy, as well as its subscales of practical, experiential, and factual knowledge, was significantly and inversely associated with preferences for LSTs, such that higher levels of death literacy and these components were modestly correlated with a lower willingness to receive aggressive LSTs. In contrast, no significant associations were found between community knowledge and preferences for any LSTs. In addition, this study indicated that death literacy may be associated with LST decision-making, with higher levels of death literacy being associated with a shift away from aggressive LSTs. These findings further support prior research suggesting that death literacy is linked to individuals’ reflection on and clarification of their own EOL care preferences.^12^ This finding provides new empirical evidence for the field of death literacy. The DLI was developed to assess EOL-related knowledge and skills embedded within the death system, comprising both private and public spheres to which individuals belong.^30^^,^^31^ Higher levels of death literacy indicate a more context-specific understanding of available care resources and the broader death system.^16^ Emerging evidence further suggests that death literacy functions as a positive sociopsychological resource, extending beyond cognitive comprehension of death-related issues to support the integration of ultimate life meaning, the reshaping of life goals, and the formation of behavioral intentions.^32^

Within the private sphere, including practical, experiential, and factual knowledge, individuals who have previously provided EOL care may acquire higher levels of practical and factual knowledge.^21^ LST-witnessing experiences have been conceptualized as situations in which individuals observe family members or peers undergoing LSTs, with such direct exposure functioning as a cognitive reference point that shapes perceptions of treatment-related risks.^33^ The potential mechanisms may be explained by social cognitive theory, which posits that witnessing LSTs, as a form of observational learning, may enhance individuals’ receptivity to death, as proximity to death can increase openness to death-related experiences.^33^^,^^34^ These experiences may foster experiential knowledge by encouraging reflection on personal values and life meaning.^17^ In contrast, the absence of a significant association between community knowledge and LST preferences may reflect contextual characteristics of Taiwan’s health care system. Community knowledge as a public sphere emphasizes two dimensions: caregiving network and support groups in the community. We must recognize the nuances in how death literacy is conceptually understood across cultures and care systems.^30^ One tentative interpretation relates to Taiwan’s hospital-centered care environment,^35^ where advanced cancer patients’ decision-making appears to rely more on personal experiences and clinical information from health care professionals.^36^ Consistent with this view, a qualitative study on emergency medical decision-making among patients with advanced cancer reported that these patients rarely sought assistance from community resources, as such resources were perceived as unable to provide the facilities and equipment considered essential for effective symptom control.^37^ These findings highlight preliminary associations between private-sphere death literacy and preferences for aggressive LSTs. Importantly, they offer a starting point for future research to clarify these relationships.

### Strengths and limitations

The present study has several strengths. First, it is the first to explore the association between death literacy and preferences for LSTs. Second, given that patients with advanced NSCLC represent a vulnerable population with poor physical status and a high symptom burden, one-to-one data collection was employed to minimize missing data and enhance data completeness.^24^ In addition, the Life-Sustaining Treatment Preferences Interview Questionnaire employed a vignette-based technique, presenting brief hypothetical scenarios to elicit participants’ views, beliefs, and attitudes toward EOL treatment decisions. This approach allows respondents to reflect from a less threatening third-person perspective,^38^ thereby reducing the need to directly relate the scenarios to their own experiences with advanced cancer. As a result, the vignette technique may help minimize psychological burden during questionnaire completion while still capturing meaningful preferences regarding LSTs. Finally, the study achieved a high participation rate of over 90%. However, because eligible participants were first invited by attending physicians, selection bias cannot be completely excluded, as patients who were more willing to discuss EOL issues may have been more likely to participate.

However, several limitations should be noted when interpreting and applying the findings. First, because the data were collected using a cross-sectional design, it is not possible to determine whether the findings are context-specific or whether preferences for LSTs remain stable over time. Second, due to the correlational nature of the study, causal relationships cannot be inferred. Third, the study was conducted at a single center, and participants were hospitalized at the time of enrollment; fatigue, illness severity, or treatment burden may have influenced their responses in ways that could not be fully accounted for. Fourth, the hypothetical scenarios presented in the questionnaire could not encompass all types of clinical circumstances encountered by patients, which would be challenging for them to imagine and make corresponding decisions. Fifth, as this study aimed to explore the association between death literacy and preferences for LSTs rather than to identify predictors, multiple regression analysis was not conducted. Therefore, potential confounding variables could not be adjusted for, and the observed associations should be interpreted with caution. Future studies should use multivariable analytical approaches to examine whether these associations remain after adjustment for relevant covariates. Finally, the sample size was relatively small, which may be attributable to challenges in recruiting patients with advanced NSCLC, who often experience poor performance status and a heavy symptom burden that restricts participation in research.

### Implications for practice and research

The present findings have important implications for clinical practice. Given that death literacy varies across populations and reflects differences in individuals’ developmental backgrounds and life experiences, incorporating the DLI into routine pre-visit assessments conducted by clinical teams may enable a systematic evaluation of patients’ competencies across domains of death literacy. For individuals identified as having low death literacy, results from this assessment can be used to pinpoint specific areas of deficiency and to guide the implementation of targeted and appropriate intervention strategies.^39^ Specifically, integrating death literacy assessment into routine clinical practice may enable earlier EOL care discussions and could support the delivery of value-concordant care.^21^ Future studies with larger and more diverse samples, including sexual and gender minority groups,^40^ and longitudinal designs or mixed-methods studies are warranted to further clarify the directionality and underlying mechanisms of these associations over time.

## Conclusions

Overall death literacy, as well as the practical, experiential, and factual knowledge subscales, was associated with preferences for LSTs in patients with advanced NSCLC. Patients in the higher death literacy group were less likely to prefer aggressive LSTs. These findings provide preliminary empirical evidence supporting a potential role of death literacy in relation to EOL-treatment preferences.

## CRediT authorship contribution statement

**G-F Lin:** contributed to conceptualization, data curation, investigation, project administration, validation, visualization, writing – original draft, and writing – review and editing. **C–Y Yang:** contributed to investigation, resources, project administration, and writing—review and editing. **Y–C Chen:** contributed to investigation, resources, project administration. **Y-L Yeh:** contributed to conceptualization and writing – review & editing. **S–C Shun:** contributed to methodology and writing – review & editing. **C–P Lin:** contributed to the conceptualization, methodology, and supervision of the study, and was involved in both the original draft writing and the review and editing of the manuscript. All authors have read and approved the final manuscript.

## Ethics statement

The study was approved by the Institutional Review Board of the National Taiwan University Hospital in Taipei City, Taiwan (Approval No. 202307012RINC) and was conducted in accordance with the 1964 Helsinki Declaration and its later amendments or comparable ethical standards. All participants provided written informed consent.

## Data availability statement

The raw data are not publicly available due to the conditions of institutional ethics approval.

## Declaration of generative AI and AI-assisted technologies in the writing process

No AI tools/services were used during the preparation of this work.

## Funding

This study was funded through a grant from the National Taiwan University Hospital (Grant No. 113-N0004). The funders had no role in considering the study design or in the collection, analysis, interpretation of data, writing of the report, or decision to submit the article for publication.

## Declaration of competing interest

The authors declare no conflict of interest.
